# Intrarater and interrater reliability of three classifications for scapular dyskinesis in athletes

**DOI:** 10.1371/journal.pone.0181518

**Published:** 2017-07-27

**Authors:** Denise Martineli Rossi, Cristiane Rodrigues Pedroni, Jaqueline Martins, Anamaria Siriani de Oliveira

**Affiliations:** 1 Department of Biomechanics, Medicine and Locomotor Apparatus Rehabilitation, Ribeirão Preto Medical School, University of São Paulo, Ribeirão Preto, São Paulo, Brazil; 2 Department of Physiotherapy and Occupational Therapy, São Paulo State University, Marília, São Paulo, Brazil; Universite de Nantes, FRANCE

## Abstract

Clinical evaluation of scapular dyskinesis (SD) aims to identify abnormal scapulothoracic movement, underlying causal factors, and the potential relationship with shoulder symptoms. The literature proposes different methods of dynamic clinical evaluation of SD, but improved reliability and agreement values are needed. The present study aimed to evaluate the intrarater and interrater agreement and reliability of three SD classifications: 1) 4-type classification, 2) Yes/No classification, and 3) scapular dyskinesis test (SDT). Seventy-five young athletes, including 45 men and 30 women, were evaluated. Raters evaluated the SD based on the three methods during one series of 8–10 cycles (at least eight and maximum of ten) of forward flexion and abduction with an external load under the observation of two raters trained to diagnose SD. The evaluation protocol was repeated after 3 h for intrarater analysis. The agreement percentage was calculated by dividing the observed agreement by the total number of observations. Reliability was calculated using Cohen Kappa coefficient, with a 95% confidence interval (CI), defined by Kappa coefficient ±1.96 multiplied by the measurement standard error. The interrater analyses showed an agreement percentage between 80% and 95.9% and an almost perfect reliability (k>0.81) for the three classification methods in all the test conditions, except the 4-type and SDT classification methods, which had substantial reliability (k<0.80) in shoulder abduction. Intrarater analyses showed agreement percentages between 80.7% and 89.3% and substantial reliability (0.67 to 0.81) for both raters in the three classifications. CIs ranged from moderate to almost perfect categories. This indicates that the three SD classification methods investigated in this study showed high reliability values for both intrarater and interrater evaluation throughout a protocol that provided SD evaluation training of raters and included several repetitions of arm movements with external load during a live assessment.

## Introduction

Scapular dyskinesis (SD) can be clinically characterized by the prominence of the medial or inferomedial border, early scapular elevation or shrugging on arm elevation, or rapid downward rotation during arm lowering [1,2]. Several factors [3–5] have been shown to be related to altered scapular position and motion. Evidence exists regarding SD in patients with shoulder conditions, such as rotator cuff injury, labral injury, and multidirectional instability [5]. However, the relationship between shoulder symptoms and SD is still unclear [2,5]. Subsequently, a comprehensive clinical evaluation of scapula is needed to understand its role in pain and to serve as a measure of impairment to guide suitable intervention programs [6].

A large number of clinical evaluation methods of scapular position and motion with different operational and methodological definitions have been reported in the literature [7–9]. Three main studies [10–12] of SD visual dynamic evaluation are suggested in the literature: 1) 4-type classification, 2) Yes/No classification, and 3) scapular dyskinesis test (SDT). The 4-type classification proposed by Kibler et al. (2002) is based on the specific kinematics of the scapula’s three-dimensional movement (Table 1), and the Yes/No classification proposed by Uhl et al. (2009) [11] considers all patterns of scapular asymmetry suggested by Kibler et al. (2002) into the “Yes” category, and the symmetric scapular motion into the “No” category (Table 1). McClure et al. (2009) [12] suggested that the SDT classifies SD based on the scapular movement disorder severity, i.e., obvious, subtle, and normal (Table 1).

**Table 1:** Original operational definitions of the classification methods of scapular dyskinesis.

**4-type [10]**	**Type 1**	Prominent inferior medial scapular border at rest and during arm motion
	**Type 2**	Prominent entire medial scapular border at rest and during arm motion
	**Type 3**	Elevation of the superior border and anterior displacement of the scapula at rest and shoulder shrug without the occurrence of significant winging of the scapula at the beginning of the movement
	**Type 4**	Both scapulae are positioned symmetrically (the scapula of the dominant member may be a bit lower) at rest and turn symmetrically upwards with the medial border attached to the thorax during movement.
**Yes/No [11]**	**Yes**	Types 1, 2, and 3: Patterns of scapular asymmetry
	**No**	Type 4: Symmetric scapular motion
**SDT [12]**	**Obvious**	Apparent prominence of any portion of the medial border or inferior angle or dysrhythmia, or excessive or premature movement of the scapula during elevation or lowering of the arm
	**Subtle**	Questionable evidence of abnormality, inconsistently present
	**Normal**	Absence of projection of the scapula, and upper and lower rotations are smooth and continuous during elevation and lowering of the arm, respectively.

Abbreviation: SDT, scapular dyskinesis test.

Overall, the methodological quality of reliability and validity studies varies from fair to poor categories [8]. The best evidence of reliability for visual dynamic evaluation methods found only two studies with good methodological quality, and still found Kappa values <0.7, which is recommended for reliability studies [9].

The 4-type classification showed moderate interrater reliability (kappa of 0.42) in asymptomatic and symptomatic subjects [10], and authors suggested that 10-min rater training, evaluating SD using video images and observing few cycles of arm elevation and lowering might have influenced reliability [10]. Uhl et al. (2009) compared reliability and validity between the Yes/No and 4-type classifications in asymptomatic and symptomatic subjects. Both classification methods showed moderate reliability, 0.41 and 0.44 Kappa, and 79% and 61% agreement, respectively. The validity of the Yes/No classification showed a higher sensitivity (74%–78%) than the 4-type (10%–54%) classification but presented decreased specificity (31%–38%) [11].

The SDT method presented moderate and substantial interrater reliability (Kappa, 0.48–0.61) in healthy overhead athletes. The same research group investigated the SDT validity through a three-dimensional electromagnetic kinematic testing while athletes performed shoulder flexion and abduction. Athletes clinically classified as having obvious dyskinesis presented less scapular upward rotation and clavicular elevation during arm elevation compared with athletes considered as having normal scapular motion [13]. The authors suggested that adding a load during arm motion helps to determine the pattern of abnormal scapular positioning [13].

A modified method of the three assessment methods evaluated in the present study was proposed by Struyf et al. (2009) [14] and evaluated in healthy musicians by separately observing tilting, winging, elevation, protraction, and rotation of the scapula [14]. This method presented better reliability only for tilt and winging (Kappa, 0.52 and 0.78, respectively), during unloaded abduction movement compared with loaded (Kappa, 0.24 and 0.50, respectively) [14].

The current “Scapular Summit” consensus recommends SD assessment by visual dynamic methods [5], but an improvement of reliability is needed to strengthen the understanding of SD on the shoulder complex [5,8]. Provided that improved reliability values can be achieved by methodological improvements suggested by the three main studies [10–12] evaluated in the present study and considering the potential lack of consensus regarding appropriate measure and reduced reliability values [8,9], this study aimed to evaluate intra- and interrater reliability of the main three SD classification methods, 4-type, Yes/No, and SDT, by using methodological improvements suggested by the original authors [10–12].

## Materials and methods

### Participants

This cross-sectional study on clinical evaluation included 75 young athletes (45 men). The participants were recruited by verbal invitation at a gymnasium during a sports event that brought together athletes from various sports. A blinded physical therapist collected the anthropometric and demographic data, number of years spent playing the current sport, hand dominance, and presence of shoulder pain via verbal interview. The convenience sample included athletes of the following sports: baseball (n = 11), judo (n = 5), taekwondo (n = 6), volleyball (n = 8), basketball (n = 21), jiu-jitsu (n = 23), and swimming (n = 1). The athletes stated that they were professional (n = 9), amateur (n = 57), and recreational (n = 9) players. All had a full range of arm elevation and lowering. Eight volunteers self-reported shoulder pain intensity in the dominant upper limb between 1 and 4 on the numeric pain scale [15]. Participants were excluded when reporting any history of shoulder surgery, scapular, humeral, or clavicular fracture, visually detectable misalignment in the thoracic spine, and systemic diseases. The study was approved by the Research Ethics Committee of São Paulo State University (protocol number 0754/2013). All study participants signed a consent form, which outlined their risks in participating in the study.

### Procedures

Two blinded physical therapists (PT_A_ and PT_B_) independently performed the SD assessment. Both physical therapists were undertaking a post-graduate degree (Masters and PhD) in musculoskeletal physical therapy and had two years of orthopedic experience. The physical therapists underwent a 9-h training session divided in three days. On the first day, the raters separately studied the original descriptions and analyzed photographs of the three SD classifications by the authors, i.e. 4-type [10], Yes/No [11], and SDT [12]. On the second day, both raters analyzed the videos presented in the McClure et al. (2009) study [12], performed the assessments without knowledge of the other outcomes, and compared and discussed the differences. On the third day, the raters independently assessed eight asymptomatic volunteers, and results were subsequently compared and discussed.

During the assessment, the participants initially stood in a relaxed position with arms at the sides, elbows straight, and shoulder in neutral rotation. Subsequently, the participants were instructed to raise both arms above his or her head simultaneously as much as possible in a 2-s period, and then lower the arms for 2 s [11,12]. The participants performed one series of 8–10 cycles (at least eight and maximum of ten) of elevation and lowering of the arms with weighted loads based on their body mass. Participants who weighed <68.1 kg and ≥68.1 kg used 1.5-kg and 2.5-kg dumbbells, respectively [12].

The raters stood approximately 2 m behind the participants with freedom to move during the test to observe the scapula from any point in the posterior frontal and sagittal planes, without performing any other evaluation, such as palpation. The raters performed the assessment simultaneously, and independently, i.e., they did not communicate their results to each other at any time, and their recordings were obscured by using a folder that covered the notes from the view of each other.

First, the raters defined the SD on Yes/No classification. Second, they chose the altered specific scapula movement (Type 1, 2, or 3) if SD was classified as “Yes.” Finally, the raters judged the degree of observed abnormal movements (obvious or subtle). If classified as “No,” it was classified accordingly as Type 4 and normal for SDT classification. Only one rater explained the test procedures to volunteers dx.doi.org/10.17504/protocols.io.imycc7w [PROTOCOL DOI].

The raters assessed both sides, but only data from the dominant upper limb were considered in this study for data reduction. After 3 h, the evaluation protocol was repeated to determine intrarater agreement and reliability.

### Statistical analysis

Data analysis was performed using Statistical Package for Social Sciences (SPSS) software (version 17, SPSS, Inc, Chicago, IL USA). The agreement percentage \was calculated by dividing the observed agreement by the total number of observations, which indicated how identical the repeated measurements were, i.e., the degree to which the raters agreed on themselves (interrater agreement), and how much each rater agreed with himself or herself (intrarater agreement) [16].

Intra- and interrater reliabilities were determined using Cohen Kappa coefficient, with its 95%, confidence interval (CI) defined by the value of the Kappa coefficient ±1.96 multiplied by the standard error of the Kappa coefficient [17]. The Cohen Kappa coefficient is recommended to determine the relative agreement between evaluators for nominal or categorical data, which eliminates the effect of the expected agreement at random [16]. The unweighted Kappa coefficient was used for nominal classification of SD based on the 4-type and Yes/No classification, and the Kappa coefficient with linear weighting was used for the ordinal classification of SD by SDT. The Kappa coefficient amplitude was 0 to 1, wherein the agreement strength followed these values:<0, poor; 0.01–0.20, slight; 0.21–0.40, fair; 0.41–0.60, moderate; 0.61–0.80, substantial; and 0.81–1, almost perfect [17].

The magnitude of the Kappa coefficient can be influenced by the prevalence index that reflects the prevalence of an attribute and by bias that is the extent to which the raters disagree on the proportion of positive (or negative) cases. Thus, these factors should be taken into account in interpreting the Kappa values [17]. The prevalence index was calculated by estimating the difference in the proportion of agreement on the positive and negative cases for the two raters (interrater reliability) and two evaluations (intrarater reliability) with values ranging from −1 to +1, where 0 indicates equal probability of positive and negative cases [18]. The bias index was calculated by estimating the difference in proportions of positive cases between the two raters (interrater reliability) or two evaluations (intrarater reliability). The absolute value of bias index ranges from 0 to 1, where 0 indicates equal marginal proportions [18]. Types 1, 2, and 3 were defined as positive cases for the 4-type classification, and obvious and subtle categories for SDT classification were defined as positive cases for index calculations.

## Results

Interrater agreement and reliability analyses of SD classification were obtained from 75 participants (Table 2). Intrarater agreement and reliability were based on analyses of 57 participants because 18 participants could not return for the second evaluation. Table 3 shows the number of participants classified with SD by each examiner and each category. Out of 75 participants, 39% to 53% were categorized with altered position or motion of the scapula during arm movements.

**Table 2:** Means of body anthropometrics, age, and sport practice time in sport (n = 75).

Characteristic	Male (n = 45)	Female (n = 30)
**Age, y**	23.7 ± 7.0	17.6 ± 5.7
**Mass, kg**	78.6 ± 13.5	62.3 ± 10.1
**Height, cm**	175.9 ± 7.3	165.6 ± 6.2
**Body mass index, kg.m**^**2**^	25.3 ± 3.8	22.8 ± 3.0
**Practice time, y**	6.8 ± 6.9	6.4 ± 6.2

Abbreviations: y, years; kg, kilogram; cm, centimeter; m, meter. The values presented as mean ± SD.

**Table 3:** Number (percentage) of participants diagnosed as having scapular dyskinesis (n = 75) by each examiner.

Classification	Test	Presence of scapular dyskinesis n (%)	
		PT_A_	PT_B_
**4-type**	**Rest**	31 (41%)	30 (40%)
	**Flex**	39 (52%)	40 (53%)
	**Abd**	30 (40%)	36 (48%)
**Yes/No**	**Rest**	31 (41%)	30 (40%)
	**Flex**	40 (53%)	39 (52%)
	**Abd**	31 (41%)	36 (48%)
**SDT**	**Rest**	30 (40%)	29 (39%)
	**Flex**	40 (53%)	39 (52%)
	**Abd**	31 (41%)	36 (48%)

Abbreviations: PT_A_, physical therapist A; PT_B_, physical therapist B; SDT, scapular dyskinesis test; Flex, flexion; Abd, abduction.

Table 4 presents the interrater agreement and reliability. The three classifications of SD presented agreement values ranging from 80% to 95.9%. Furthermore, all classifications presented almost perfect reliability (*k* > 0.81), except the 4-type and SDT classifications obtained in the abduction movement, which showed substantial reliability. The confidence intervals showed a reliability substantial and almost perfect for Yes/No and SDT, and moderate (*k* < 0.61) for the 4-type classification.

**Table 4:** Interrater agreement and reliability of the three classifications of scapular dyskinesis (n = 75).

		P (%)	Kappa Coefficient (95% CI)	Prevalence Index	Bias Index
**4-type**	**Rest**	91.8	0.85 (0.73–0.96)	-0.23	0.01
	**Flex**	89.3	0.81 (0.69–0.93)	0.01	0.00
	**Abd**	84.0	0.71 (0.58–0.85)	-0.17	0.07
**Yes/No**	**Rest**	95.9	0.91 (0.82–1.00)	-0.19	0.01
	**Flex**	95.0	0.89 (0.79–1.00)	0.07	0.00
	**Abd**	90.7	0.81 (0.68–0.94)	-0.11	0.07
**SDT**	**Rest**	90.4	0.85 (0.74–0.95)	-0.25	0.01
	**Flex**	89.2	0.86 (0.76–0.95)	0.00	0.00
	**Abd**	80.0	0.74 (0.61–0.86)	-0.21	0.06

Abbreviations: P, percentage of agreement; CI, confidence interval; SDT, scapular dyskinesis test; Flex, flexion; Abd, abduction.

For intrarater agreement, raters PT_A_ and PT_B_ presented relatively high percentage values of agreement between the two measurements for the three classifications of SD, ranging from 82.1% to 89.3% and from 80.7% to 88.9%, respectively (Table 5). Intrarater reliability for both raters, on the three classifications, was substantial (0.67–0.81). However, the CIs indicated that reliability for the population was between moderate and almost perfect categories (Table 5).

**Table 5:** Intrarater agreement and reliability of the three classifications of scapular dyskinesis (n = 57) by two examiners.

	P (%)		Kappa (95% CI)		Prevalence index		Bias index	
	PT_A_	PT_B_	PT_A_	PT_B_	PT_A_	PT_B_	PT_A_	PT_B_
**4-type**								
**Rest**	82.1	87.0	0.67 (0.50–0.85)	0.76 (0.60–0.92)	-0.25	-0.20	0.03	0.00
**Flex**	87.5	84.7	0.79 (0.65–0.94)	0.71 (0.54–0.88)	0.02	-0.07	0.03	0.00
**Abd**	84.2	84.2	0.68 (0.50–0.87)	0.69 (0.51–0.87)	-0.35	-0.25	0.05	0.03
**Yes/No**								
**Rest**	85.7	88.9	0.70 (0.52–0.88)	0.77 (0.60–0.94)	-0.21	-0.19	0.04	0.00
**Flex**	89.3	85.7	0.79 (0.63–0.95)	0.71 (0.53–0.90)	0.04	-0.05	0.04	0.00
**Abd**	87.7	86.0	0.73 (0.54–0.91)	0.70 (0.52–0.89)	-0.32	-0.23	0.05	0.04
**SDT**								
**Rest**	83.9	88.9	0.69 (0.51–0.87)	0.79 (0.64–0.95)	-0.23	-0.19	0.03	0.00
**Flex**	83.9	82.1	0.81 (0.70–0.93)	0.74 (0.58–0.89)	-0.02	-0.11	0.03	0.00
**Abd**	84.2	80.7	0.76 (0.61–0.91)	0.66 (0.48–0.85)	-0.35	-0.25	0.00	0.02

Abbreviations: P, percentage of agreement; CI, confidence interval; SDT, scapular dyskinesis test; Flex, flexion; Abd, abduction.

The prevalence index for both inter- and intrarater reliabilities showed mostly small negative values, i.e., no prevalence effect. Slightly larger values for rest and abduction movement occurred because of higher proportion of agreement on negative cases, but the prevalence index was still small (Tables 4 and 5). The bias index presented values very close to zero, indicating no difference in proportions of positive cases between the two examiners (Table 4) or between the two evaluations of each examiner (Table 5).

## Discussion

In this study, inter- and intrarater reliabilities were almost perfect and substantial, respectively, for the three SD classification methods namely: 1) 4-type classification, 2) Yes/No classification, and 3) SDT. The CI values of the Kappa coefficient that enables a statistical inference for the population encompassed the substantial and almost perfect categories of interrater reliability for the SDT and Yes/No classifications, and moderate category for the 4-type classification. For intrarater analysis, CIs covered moderate reliability for the three SD classification methods.

Several elements highlighted in previous studies for improving agreement were applied in the present study. The present study used the suggestion by Uhl et al. (2009) [11] to increase the number of arm elevation and lowering cycles used to observe SD and the suggestion by McClure et al. (2009) [12] to add resistance (external load) to the movement. Furthermore, as Kibler et al. (2002) [10] proposed, our raters were previously trained by using the authors’ original operational definitions and the photos and videos compiled by McClure et al. (2009) [12] that demonstrated normal and abnormal scapular positioning and scapulothoracic movements.

The original studies demonstrated methods with moderate reliability to classify overall SD [10–12]. The 4-type classification presented moderate inter- (Kappa = 0.42) and intrarater (Kappa = 0.49) reliabilities among physical therapists using a videotape in asymptomatic and symptomatic subjects [10]. The same result was found for Yes/No classification (Kappa = 0.41), with an agreement percentage of 79% between raters in asymptomatic and symptomatic subjects [11]. The SDT classification presented moderate and substantial interrater reliabilities (Kappa, 0.48–0.61) and agreement percentage from 75% to 82%, based on live evaluation and videotape [12] in healthy overhead athletes.

Huang et al. (2015) [19] added the possibility of visual observation and manual palpation combined for an easier SD evaluation on detecting the posterior displacement of scapular inferior angle or medial border during arm movements in subjects with shoulder pain. The authors also suggested the inclusion of mixed pattern SD classification (e.g., types 1 and 2) in the same scapula. This modified method resulted in a reliability that ranged from moderate to substantial (Kappa, 0.57–0.64) during the arm-lowering phase. These authors also investigated scapular kinematics during arm motions between patients with shoulder pain with and without SD. Findings revealed that patients classified with type 2 and mixed pattern presented increased scapular internal rotation during the arm-lowering phase, and patients classified with type 1 showed increased scapular anterior tilt compared with patients without SD (type 4) [20]. The present study did not include the modified method proposed by Huang et al. (2015) because it aimed to evaluate the main three SD classification methods and also because palpation could not be simultaneously performed by the two raters.

Comparing the three classification methods evaluated in the present study, only the Yes/No classification showed an almost perfect interrater reliability during rest, flexion, and abduction. This finding may be because the “Yes” rating is more inclusive and does not limit the rater to selecting a single plane to observe the change. Uhl et al. (2009) investigated the validity of the Yes/No classification where they found that sensitivity and specificity ranged from 74% to 78% and from 31% to 38% for scaption and shoulder flexion, respectively [11]. Therefore, this classification better identifies the true presence of SD but is less specific and suggests a risk of classifying participants with SD when SD is absent [11].

Our findings suggested that SDT and 4-type classification presented a slightly less interrater reliability during abduction test compared with rest and shoulder flexion. These decreased Kappa values might have been affected by two factors: prevalence index and agreement percentage. The increased prevalence index means that raters agreed more that participants did not have SD (38/75 observations for the 4-type classification; 38/75 observations for SDT) than that they had SD (25/75 observations for the 4-type classification; 22/75 observations for SDT) during shoulder abduction. This result is consistent with the previous study, wherein raters observed obvious dyskinesis more frequently during shoulder flexion (91/284 observations) than during shoulder abduction (54/284 observations) [12]. The reduced agreement percentage (84% for the 4-type classification; 80% for SDT) during abduction compared with shoulder flexion (89% for the 4-type classification; 89% for SDT) might also have affected Kappa values.

Previous studies used video recordings of the scapular motion taped from the back of the participants to determine the intra- [10] and interrater reliabilities [10,12]. McClure et al. (2009) [12] used live rating, but the examiners were allowed to be aware of the judgments of the other raters [12]. The “Guidelines for reporting reliability and agreement studies” [16] recommends that no communication should be allowed in studies where raters simultaneously conduct an evaluation. The present study protocol included live rating because it allowed viewing the scapula from more angles than those available in videotapes; raters were aware they could not discuss their classifications.

Several factors make SD challenging in the clinical context, such as high variability of scapular movement, possibility of adaptive strategies, difficulty of a “normal” pattern definition, poor methodological quality regarding properties, and lack of clarity in the relationship between SD and presence of symptoms [9,21]. Based on the findings of the present study, we recommend to add at least eight repetitions of shoulder movements with external load based on body mass (1.5 kg or 2.5 kg) and an SD evaluation training (SD description study, photographs and videos analyzed, and pilot assessment) of physical therapists that do not need to be an expert in the field. The present study added load during arm motions based on McClure et al. (2009) [12]. A modified method proposed by Struyf et al. (2009) [14] presented better reliability during unloaded shoulder abduction compared with loaded movement using a different classification method. Adding load to the movement seems to improve the reliability of SD classification depending on the evaluation method. More information is required regarding adding load to the movement and also further research into new approaches to scapular evaluation based on whole system and context instead of the scapula alone [21].

Similar to the previous videotape methods, this study has a limitation because it does not consider the scapulothoracic variability found in real clinical situations when two or more examiners assess patients in different moments. Another limitation is that the sample profile included only athletes without self-report of shoulder pain present during the assessment. More information is required regarding the SD judgment as proposed in this study in patients with pain or range of motion limitation. Two strengths of this study included a comparison of the three SD classifications using the same protocol, and the protocol that considered the recommendations of each author to improve SD identification.

## Conclusions

The SDT, Yes/No, and 4-type SD classification methods investigated in the present study showed high reliability values for both intra- and interrater assessments throughout a protocol, including training of the raters, multiple repetitions of the arm movements, and external load in a live setting.

## Supporting information

S1 Tables Contingency tables of interrater reliability. Abbreviations: PT, physical therapist; SDT, scapular dyskinesis test.
(DOCX)

S2 Tables Contingency tables of PT_A_ intrarater reliability. Abbreviations: PT, physical therapist; SDT, scapular dyskinesis test.
(DOCX)

S3 Tables Contingency tables of PT_B_ intrarater reliability. Abbreviations: PT, physical therapist; SDT, scapular dyskinesis test.
(DOCX)

## References

[1] Kibler WB, Sciascia A. Current concepts: scapular dyskinesis. Br J Sports Med. 2010;44: 300–305. doi: 10.1136/bjsm.2009.058834 19996329 10.1136/bjsm.2009.058834

[2] Kibler WB, Sciascia A, Wilkes T. Scapular dyskinesis and its relation to shoulder injury. J Am Acad Orthop Surg. 2012;20: 364–72. doi: 10.5435/JAAOS-20-06-364 22661566 10.5435/JAAOS-20-06-364

[3] Kebaetse M, McClure P, Pratt NA. Thoracic position effect on shoulder range of motion, strength, and three-dimensional scapular kinematics. Arch Phys Med Rehabil. 1999;80: 945–50. 10453773 10.1016/s0003-9993(99)90088-6

[4] Kibler WB. Scapular Dysfunction. J Athl Train. 2006;11: 6–9.

[5] Kibler WB, Ludewig PM, McClure PW, Michener LA, Bak K, Sciascia AD. Clinical implications of scapular dyskinesis in shoulder injury: the 2013 consensus statement from the “Scapular Summit”. Br J Sports Med. 2013;47: 877–85. doi: 10.1136/bjsports-2013-092425 23580420 10.1136/bjsports-2013-092425

[6] Wright AA, Wassinger CA, Frank M, Michener LA, Hegedus EJ. Diagnostic accuracy of scapular physical examination tests for shoulder disorders: a systematic review. Br J Sorts Med. 2013;47: 886–892. 10.1136/bjsports-2012-091573 23080313

[7] Struyf F, Nijs J, Mottram S, Roussel NA, Cools AM, Meeusen R. Clinical assessment of the scapula: a review of the literature. Br J Sports Med. 2014;48: 883–890. doi: 10.1136/bjsports-2012-091059 22821720 10.1136/bjsports-2012-091059

[8] Larsen CM, Juul-Kristensen B, Lund H, Sogaard K. Measurement properties of existing clinical assessment methods evaluating scapular positioning and function. A systematic review. Physiother Therory Pract. 2014;30: 452–482. 10.3109/09593985.2014.899414 24678755

[9] D’hondt NE, Kiers H, Pool JJ, Hacquebord ST, Terwee CB, Veeger DH. Reliability of performance-based clinical measurements to assess shoulder girdle kinematic and positioning: A systematic review. Phys Ther. 2016;96. 10.2522/ptj.20160088 27587801

[10] Kibler WB, Uhl TL, Maddux JW, Brooks PV, Zeller B, McMullen J. Qualitative clinical evaluation of scapular dysfunction: a reliability study. J Shoulder Elb Surg. 2002;11: 550–556. 10.1067/mse.2002.126766 12469078

[11] Uhl TL, Kibler WB, Gecewich B, Tripp BL. Evaluation of clinical assessment methods for scapular dyskinesis. Arthroscopy. 2009;25: 1240–1248. doi: 10.1016/j.arthro.2009.06.007 19896045 10.1016/j.arthro.2009.06.007

[12] McClure P, Tate AR, Kareha S, Irwin D, Zlupko E. A clinical method for identifying scapular dyskinesis, part 1: reliability. J Athl Train. 2009;44: 160–164. doi: 10.4085/1062-6050-44.2.160 19295960 10.4085/1062-6050-44.2.160 PMC2657031

[13] Tate AR, McClure P, Kareha S, Irwin D, Barbe MF. A clinical method for identifying scapular dyskinesis, Part 2: Validity. J Athl Train. 2009; 44: 160–164. doi: 10.4085/1062-6050-44.2.160 19295961 10.4085/1062-6050-44.2.165 PMC2657032

[14] Struyf F, Nijs J, Coninck KD, Giunta M, Mottram S, Meeusen R. Clinical assessment of scapular positioning in musicians: An intertester reliability study. J Athl Train. 2009; 44: 519–526. doi: 10.4085/1062-6050-44.5.519 19771291 10.4085/1062-6050-44.5.519 PMC2742462

[15] Dworkin RH, Turk DC, Farrar JT, Haythornthwaite JA, Jensen MP, Katz NP, et al Core outcome measures for chronic pain clinical trials: IMMPACT recommendations. Pain. 2005;113: 9–19. doi: 10.1016/j.pain.2004.09.012 15621359 10.1016/j.pain.2004.09.012

[16] Kottner J, Audigé L, Brorson S, Donner A, Gajewski BJ, Hróbjartsson A, et al Guidelines for Reporting Reliability and Agreement Studies (GRRAS) were proposed. J Clin Epidemiol. 2011;64: 96–106. doi: 10.1016/j.jclinepi.2010.03.002 21130355 10.1016/j.jclinepi.2010.03.002

[17] Sim J, Wright CC. The kappa statistic in reliability studies: use, interpretation, and sample size requirements. Phys Ther. 2005;85: 257–68. 15733050

[18] Byrt T, Bishop J, Carlin JB. Bias, prevalence and kappa. J Clin Epidemiol. 1993;46: 234–429. 10.1016/0895-4356(93)90018-v 8501467

[19] Huang T, Huang H, Wang T, Tsai Y, Lin J. Comprehensive classification test of scapular dyskinesis: A reliability study. Manual Therapy. 2015;20: 427–432. doi: 10.1016/j.math.2014.10.017 25435131 10.1016/j.math.2014.10.017

[20] Huang T, Ou H, Huang C, Lin J. Specific kinematics and associated muscle activation in individuals with scapular dyskinesis. J Shoulder Surg. 2015; 24: 1227–1234. 10.1016/j.jse.2014.12.022 25704212

[21] Willmore EG, Smith MJ. Scapular dyskinesia: evolution towards a systems-based approach. Shoulder & Elbow. 2016; 8: 61–70. 27583003 10.1177/1758573215618857 PMC4935167

