# Supplementary material for: Intrarater and interrater reliability of three classifications for scapular dyskinesis in athletes
Source: PLoS One. 2017 Jul 27;12(7):e0181518. doi: 10.1371/journal.pone.0181518 (PMC5531566; doi:10.1371/journal.pone.0181518)
Supplement: S1 Tables — Abbreviations: PT, physical therapist; SDT, scapular dyskinesis test. (DOCX) [file pone.0181518.s001.docx]

**S1 Tables. Contingency tables of interrater reliability.** Abbreviations: PT, physical therapist; SDT, scapular dyskinesis test.

**4-Types classification method during rest.**

|  |  | **PT _B_** |  |  |  |  |
| --- | --- | --- | --- | --- | --- | --- |
|  |  | **Type 1** | **Type 2** | **Type 3** | **Type 4** | **Total** |
| **PT _A_** | **Type 1** | 21 | 0 | 0 | 1 | 22 |
|  | **Type 2** | 3 | 4 | 0 | 1 | 8 |
|  | **Type 3** | 0 | 0 | 0 | 0 | 0 |
|  | **Type 4** | 1 | 0 | 0 | 42 | 43 |
|  | **Total** | 25 | 4 | 0 | 44 | 73 |

**4-Types classification method during flexion.**

|  |  | **PT _B_** |  |  |  |  |
| --- | --- | --- | --- | --- | --- | --- |
|  |  | **Type 1** | **Type 2** | **Type 3** | **Type 4** | **Total** |
| **PT _A_** | **Type 1** | 32 | 1 | 0 | 1 | 34 |
|  | **Type 2** | 3 | 2 | 0 | 1 | 6 |
|  | **Type 3** | 0 | 0 | 0 | 0 | 0 |
|  | **Type 4** | 2 | 0 | 0 | 33 | 35 |
|  | **Total** | 37 | 3 | 0 | 35 | 75 |

**4-Types classification method during abduction.**

|  |  | **PT _B_** |  |  |  |  |
| --- | --- | --- | --- | --- | --- | --- |
|  |  | **Type 1** | **Type 2** | **Type 3** | **Type 4** | **Total** |
| **PT _A_** | **Type 1** | 23 | 1 | 0 | 0 | 24 |
|  | **Type 2** | 4 | 2 | 0 | 1 | 7 |
|  | **Type 3** | 0 | 0 | 0 | 0 | 0 |
|  | **Type 4** | 6 | 0 | 0 | 38 | 44 |
|  | **Total** | 33 | 3 | 0 | 39 | 75 |

**Yes/No classification method during rest**.

|  |  | **PT _B_** |  |  |
| --- | --- | --- | --- | --- |
|  |  | **Yes** | **No** | **Total** |
| **PT _A_** | **Yes** | 28 | 2 | 30 |
|  | **No** | 1 | 42 | 43 |
|  | **Total** | 29 | 44 | 73 |

**Yes/No classification method during flexion.**

|  |  | **PT _B_** |  |  |
| --- | --- | --- | --- | --- |
|  |  | **Yes** | **No** | **Total** |
| **PT _A_** | **Yes** | 37 | 2 | 39 |
|  | **No** | 2 | 33 | 35 |
|  | **Total** | 39 | 35 | 74 |

**Yes/No classification method during abduction.**

|  |  | **PT _B_** |  |  |
| --- | --- | --- | --- | --- |
|  |  | **Yes** | **No** | **Total** |
| **PT _A_** | **Yes** | 30 | 1 | 31 |
|  | **No** | 6 | 38 | 44 |
|  | **Total** | 36 | 39 | 75 |

**SDT classification method during rest.**

|  |  | **PT _B_** |  |  |  |
| --- | --- | --- | --- | --- | --- |
|  |  | **Obvious** | **Subtle** | **Normal** | **Total** |
| **PT _A_** | **Obvious** | 4 | 2 | 0 | 6 |
|  | **Subtle** | 2 | 20 | 1 | 23 |
|  | **Normal** | 0 | 2 | 42 | 44 |
|  | **Total** | 6 | 24 | 43 | 73 |

**SDT classification method during flexion.**

|  |  | **PT _B_** |  |  |  |
| --- | --- | --- | --- | --- | --- |
|  |  | **Obvious** | **Subtle** | **Normal** | **Total** |
| **PT _A_** | **Obvious** | 28 | 1 | 0 | 29 |
|  | **Subtle** | 3 | 5 | 2 | 10 |
|  | **Normal** | 2 | 0 | 33 | 35 |
|  | **Total** | 33 | 6 | 35 | 74 |

**SDT classification method during flexion.**

|  |  | **PT _B_** |  |  |  |
| --- | --- | --- | --- | --- | --- |
|  |  | **Obvious** | **Subtle** | **Normal** | **Total** |
| **PT _A_** | **Obvious** | 18 | 6 | 2 | 26 |
|  | **Subtle** | 2 | 4 | 4 | 10 |
|  | **Normal** | 1 | 0 | 38 | 39 |
|  | **Total** | 21 | 10 | 44 | 75 |
