# Supplementary material for: Intrarater and interrater reliability of three classifications for scapular dyskinesis in athletes
Source: PLoS One. 2017 Jul 27;12(7):e0181518. doi: 10.1371/journal.pone.0181518 (PMC5531566; doi:10.1371/journal.pone.0181518)
Supplement: S2 Tables — Abbreviations: PT, physical therapist; SDT, scapular dyskinesis test. (DOCX) [file pone.0181518.s002.docx]

**S2 Tables. Contingency tables of PT_A_ intrarater reliability.** Abbreviations: PT, physical therapist; SDT, scapular dyskinesis test.

**4-Types classification method during rest.**

| **PT_A_** |  | **First** |  |  |  |  |
| --- | --- | --- | --- | --- | --- | --- |
|  |  | **Type 1** | **Type 2** | **Type 3** | **Type 4** | **Total** |
| **Second** | **Type 1** | 11 | 2 | 0 | 2 | 15 |
|  | **Type 2** | 0 | 5 | 0 | 1 | 6 |
|  | **Type 3** | 0 | 0 | 0 | 0 | 0 |
|  | **Type 4** | 3 | 2 | 0 | 30 | 35 |
|  | **Total** | 14 | 9 | 0 | 33 | 56 |

**4-Types classification method during flexion.**

| **PT_A_** |  | **First** |  |  |  |  |
| --- | --- | --- | --- | --- | --- | --- |
|  |  | **Type 1** | **Type 2** | **Type 3** | **Type 4** | **Total** |
| **Second** | **Type 1** | 19 | 0 | 0 | 3 | 22 |
|  | **Type 2** | 1 | 5 | 0 | 1 | 7 |
|  | **Type 3** | 0 | 0 | 1 | 0 | 1 |
|  | **Type 4** | 2 | 0 | 0 | 24 | 26 |
|  | **Total** | 22 | 5 | 1 | 28 | 56 |

4**-Types classification method during abduction.**

| **PT_A_** |  | **First** |  |  |  |  |
| --- | --- | --- | --- | --- | --- | --- |
|  |  | **Type 1** | **Type 2** | **Type 3** | **Type 4** | **Total** |
| **Second** | **Type 1** | 10 | 2 | 0 | 2 | 14 |
|  | **Type 2** | 0 | 3 | 0 | 0 | 3 |
|  | **Type 3** | 0 | 0 | 1 | 0 | 1 |
|  | **Type 4** | 5 | 0 | 0 | 34 | 39 |
|  | **Total** | 15 | 5 | 1 | 36 | 57 |

**Yes/No** **classification method during rest.**

| **PT_A_** |  | **First** |  |  |
| --- | --- | --- | --- | --- |
|  |  | **Yes** | **No** | **Total** |
| **Second** | **Yes** | 18 | 3 | 21 |
|  | **No** | 5 | 30 | 35 |
|  | **Total** | 23 | 33 | 56 |

**Yes/No** **classification method during flexion.**

| **PT_A_** |  | **First** |  |  |
| --- | --- | --- | --- | --- |
|  |  | **Yes** | **No** | **Total** |
| **Second** | **Yes** | 26 | 4 | 30 |
|  | **No** | 2 | 24 | 26 |
|  | **Total** | 28 | 28 | 56 |

**Yes/No classification method during abduction.**

| **PT_A_** |  | **First** |  |  |
| --- | --- | --- | --- | --- |
|  |  | **Yes** | **No** | **Total** |
| **Second** | **Yes** | 16 | 2 | 18 |
|  | **No** | 5 | 34 | 39 |
|  | **Total** | 21 | 36 | 57 |

**SDT classification method during rest.**

| **PT_A_** |  | **First** |  |  |  |
| --- | --- | --- | --- | --- | --- |
|  |  | **Obvious** | **Subtle** | **Normal** | **Total** |
| **Second** | **Obvious** | 1 | 0 | 0 | 1 |
|  | **Subtle** | 1 | 16 | 3 | 20 |
|  | **Normal** | 0 | 5 | 30 | 35 |
|  | **Total** | 2 | 21 | 33 | 56 |

**SDT classification method during flexion.**

| **PT_A_** |  | **First** |  |  |  |
| --- | --- | --- | --- | --- | --- |
| **PT_A_** |  | **Obvious** | **Subtle** | **Normal** | **Total** |
| **Second** | **Obvious** | 21 | 1 | 1 | 23 |
|  | **Subtle** | 2 | 2 | 3 | 7 |
|  | **Normal** | 0 | 2 | 24 | 26 |
|  | **Total** | 23 | 5 | 28 | 56 |

**SDT classification method during abduction.**

| **PT_A_** |  | **First** |  |  |  |
| --- | --- | --- | --- | --- | --- |
|  |  | **Obvious** | **Subtle** | **Normal** | **Total** |
| **Second** | **Obvious** | 12 | 1 | 1 | 14 |
|  | **Subtle** | 1 | 2 | 1 | 4 |
|  | **Normal** | 1 | 4 | 34 | 39 |
|  | **Total** | 14 | 7 | 36 | 57 |
